# Omeprazole Minimally Alters the Fecal Microbial Community in Six Cats: A Pilot Study

**DOI:** 10.3389/fvets.2018.00079

**Published:** 2018-04-16

**Authors:** Sarah M. Schmid, Jan S. Suchodolski, Josh M. Price, M. K. Tolbert

**Affiliations:** ^1^Department of Small Animal Clinical Sciences, College of Veterinary Medicine, The University of Tennessee, Knoxville, TN, United States; ^2^Gastrointestinal Laboratory, Department of Small Animal Clinical Sciences, College of Veterinary Medicine and Biomedical Sciences, Texas A&M University, College Station, TX, United States

**Keywords:** 16S ribosomal RNA gene sequencing, feline, proton pump inhibitor, omeprazole, microbiota

## Abstract

Although they have historically been thought of as safe medications, proton pump inhibitors such as omeprazole have been associated with an increased risk of enteric, particularly *Clostridium difficile*, infections in people. In cats, omeprazole is often the first choice acid suppressant prescribed for the treatment of upper gastrointestinal (GI) ulceration and bleeding. Despite this, no studies to date have explored the effect of omeprazole on the feline fecal microbiome and metabolome. Therefore, the purpose of this pilot study was to evaluate the effect of prolonged omeprazole administration on the fecal microbiome and metabolome in healthy cats to identify targets for analysis in a larger subset of cats with GI disease. A within-subjects, before and after, pilot study was performed whereby six healthy adult cats received 60 days of placebo (250 mg lactose PO q 12 h) followed by 5 mg (0.83–1.6 mg/kg PO q 12 h) omeprazole. On days 0, 30, and 60 of placebo and omeprazole therapy, the fecal microbiome and metabolome were characterized utilizing 16S ribosomal RNA sequencing by Illumina and untargeted mass spectrometry-based methods, respectively. Omeprazole administration resulted in no significant changes in the global microbiome structure or richness. However, transient changes were noted in select bacterial groups with omeprazole administration resulting in an increased sequence percentage of *Streptococcus, Lactobacillus, Clostridium*, and *Faecalibacterium* spp. and a decreased sequence percentage of *Bifidobacterium* spp. Significance was lost for all of these bacterial groups after adjustment for multiple comparisons. The fecal concentration of *O*-acetylserine and aminomalonate decreased with omeprazole therapy, but significance was lost after adjustment for multiple comparisons. The results of this pilot study conclude that omeprazole has a mild and transient impact on the fecal microbiome and metabolome when orally administered to healthy cats for 60 days. Based on the findings of this pilot study, evaluation of the effect of omeprazole specifically on *Streptococcus, Lactobacillus, Clostridium, Faecalibacterium*, and *Bifidobacterium* spp. is warranted in cats with primary GI disease.

## Introduction

Shown to be superior to histamine-2 receptor antagonists in raising gastric pH in cats, proton pump inhibitors (PPIs) are often the first choice acid suppressant prescribed to cats with diseases suspected to result in ulcerative esophagitis, excessive gastric acidity, and gastrointestinal (GI) bleeding (1, 2). PPIs have historically been thought of as relatively safe drugs; however, recent reports in people suggest their association with a wide range of disorders such as cobalamin deficiency, osteoporosis-related fractures, and community-acquired pneumonia (3–5).

Gastric acid production serves as a first line of defense against ingested acid-sensitive infectious agents (6). Consequently, PPI-induced increases in gastric pH might also result in gastric bacterial overgrowth and development of enteric infections (7). Indeed, in people, PPI use has been associated with an increased risk of enteric *Clostridium difficile* infections (8–10) and small intestinal bacterial overgrowth (11). There are few veterinary studies evaluating the effect of PPIs on the microbiome. Evaluating the colonic contents from rats, Kanno et al. showed that oral administration of omeprazole resulted in a dose-dependent increase in all intestinal bacteria, with the exception of *Bifidobacterium* (12). The effect of omeprazole on the GI microbiota has also recently been evaluated in dogs. Twice-daily administration of omeprazole for 15 days increased fecal *Lactobacillus* in all study dogs and decreased *Faecalibacterium* and the *Bacteroidetes*–*Prevotella*–*Prophyromonas* group in male dogs with the most significant effects noted in the stomach and small intestine (13). Decreases in fecal *Faecalibacterium* also occur in people receiving PPIs (14). Despite the widespread use of PPIs in cats, no studies have investigated their effect on the composition of the feline microbiome.

Previous culture-independent 16S ribosomal RNA (rRNA) analysis of healthy feline fecal samples revealed that the Firmicutes phylum predominates, followed by Proteobacteria, Bacteroidetes, Fusobacteria, and Actinobacteria, respectively (15–17). Although these findings are similar to those identified in dogs, cats have greater numbers of anaerobic bacteria in their small intestine compared with their canine counterpart (18–20). In addition, cats are “obligate” carnivores and their diet is composed of primarily animal-based protein, supplemented with plant-based fibrous material (21). These differences suggest that the feline fecal microbiome may respond differently to chronic PPI administration than dogs.

Alteration in the microbiota can result in changes in the relative concentrations of small molecular metabolites, including lipids, sugars, and amino acids. Consequently, evaluation of metabolomics in conjunction with the microbiome can provide a functional overview of biochemical processes that can be altered as a result of PPI administration (22). For instance, in people omeprazole therapy results in increased lactate, which might be the result of overgrowth of *Streptococcus* spp. which produce lactate through fermentation (23, 24). Overgrowth of lactate-producing bacteria has also been shown to occur with omeprazole administration in rodents (12). To date, no veterinary studies have evaluated the effect of PPIs on the feline fecal metabolome.

The aforementioned human and canine studies raise concern that prolonged PPI therapy might not be safe in cats; however, to date, the effects of PPI administration on the composition of the microbiota and metabolome in the feces of cats have not been evaluated. The central objective of this study was to evaluate the effect of chronic omeprazole administration on the fecal bacterial microbiome and metabolome of healthy cats. Based on previous findings in people, rats, and dogs, we hypothesized that oral omeprazole administration would result in a decrease in fecal *Faecalibacterium* and *Helicobacter* spp. and an increase in the *Lactobacillus* and *Clostridium* groups in healthy cats.

## Materials and Methods

### Cats

This study included six adult domestic shorthair cats that were part of a previously published study that evaluated the effect of chronic oral omeprazole administration on serum calcium, magnesium, cobalamin, and gastrin concentrations and bone mineral density in cats (25). Six cats were included in the pilot study as this is the suggested minimum number of patients necessary to perform pharmacological studies (26). The Institutional Animal Care and Use Committee at the University of Tennessee approved the protocol for this study (32312-0115). The study subjects included three spayed female and three neutered male cats, aged 7–10 years (median, 8 years) with a median weight of 4.14 kg (3.22–5.46 kg). The cats were determined to be healthy before study enrollment on the basis of an unremarkable medical history and normal physical examination, blood work (complete blood count, serum chemistry, TT4), and urinalysis. All cats were fed a maintenance diet (Hill’s Science Diet, Hill’s Nutrition, Topeka, KS, USA) before, during, and following the study period. Cats that received antibiotics were excluded from study enrollment. However, a cat that received metronidazole from day 14 to day 16 of omeprazole therapy was included on the basis that the microbiome of dogs has been shown to return to normal 2 weeks after metronidazole (Flagyl, Pfizer Inc., New York, NY, USA) administration (27). The cat developed diarrhea, a common side effect of omeprazole, on day 14 of omeprazole therapy and the diarrhea quickly resolved with administration of metronidazole. Since the next stool sample was collected 2 weeks after discontinuation of the metronidazole on day 30, it was deemed unlikely to affect the results seen at day 30, and the cat was included. Another cat that received amoxicillin trihydrate/clavulanate potassium (Clavamox Drops, Zoetis Services LLC., Parsippany, NJ, USA) on day 10 to day 24 of placebo was included as no significant differences were appreciated between day 0 and day 30 of placebo. Appetite and activity did not change before, during, and after antibiotic therapy for either cat.

### Study Design and Fecal Sample Collection

A within-subjects, before and after, study was performed whereby all cats received 60 days of consecutive treatment with placebo (250 mg lactose encapsulated in size #3 gelatin capsule, Spectrum Chemical Mfg Corp., Gardena, CA, USA) *per os* q 12 h, followed by 5 mg (0.83–1.6 mg/kg) *per os* q 12 h omeprazole (Dexcel Pharma Technologies Ltd., Yokneam, Israel) after a 4-day rest period (25). A before and after study was performed rather than a crossover design given that the necessary washout period for omeprazole administration is unknown. On days 0 (“baseline”), 30, and 60 of placebo and omeprazole therapy, free catch fecal samples were collected from each of the cats. The samples were collected in the morning and within 12 h of defecation. Storage at room temperature for up to 24 h before sample analysis has been shown to minimal effect on the integrity of extracted nuclei acid and the composition of the microbial community in both human and feline stool samples (28, 29). Following collection, fecal samples were stored at −80°C until analysis.

### DNA Extraction and 16S rRNA Sequencing

Extraction, sequencing, and analysis of microbiome and metabolome data were performed similarly to that previously described (30). Briefly, genomic DNA was extracted from 100 mg aliquots of feces using a commercially available extraction kit (PowerSoil^®^, Mo Bio Laboratories, Carlsbad, CA, USA) following the manufacturer’s instructions. Illumina sequencing of the bacterial 16S rRNA genes was performed using primers 515F (5′-GTGCCAGCMGCCGCGGTAA-3′) to 806R (5′-GGACTACVSGGGTATCTAAT-3′) at the MR DNA laboratory (www.mrdnalab.com, Shallowater, TX, USA).

### Analysis of 16S rRNA Genes

Raw sequence data were screened, trimmed, filtered, and barcoded as well as chimera sequences depleted from the dataset using QIIME v1.8.0 pipeline (31) and UCHIME (32). Operational taxanomic units (OTUs) were assigned based on at least 97% sequence similarity against the Greengenes reference database (33). Sequence information is available through the NCBI GenBank database within a short read archive under accession SRP097213.

Alpha diversity was measured with the Chao1 (richness), Shannon diversity, and observed OTU metrics. Beta diversity was evaluated with the phylogeny based weighted UniFrac (34) distance metric and visualized *via* principal coordinate analysis plots.

### Quantitative Real-Time PCR (qPCR)

In an effort to support the findings of Illumina sequencing, fecal bacterial communities that exhibited a significant change in percent sequence with omeprazole treatment were further evaluated by qPCR. The abundances of *Clostridium perfringens, Clostridium hiranonis, Clostridium difficile*, and *Bifidobacterium* spp. were estimated by qPCR in the obtained fecal DNA samples using published oligonucleotides. The qPCR cycling, primer oligonucleotide sequences, and respective annealing temperatures for selected bacterial groups have been previously described (13, 30, 35, 36). The qPCR data were expressed as log amount of DNA (fg) for each bacterial group per 10 ng of isolated total DNA.

### Metabolomics

Fecal samples were stored at −80°C until shipped on dry ice for preparation and analysis by the West Coast Metabolomics Center (University of California, Davis, CA, USA). Fecal samples in aliquots of approximately 10 mg underwent homogenization and extraction. Following centrifugation, dried supernatant was resuspended in methanol/chloroform, and internal standards were added. Samples were derivatized by methoxyamine hydrochloride in pyridine and *N*-methyl-*N*-trimethylsilyltrifluoroacetamide as previously described (30). The fecal metabolome was characterized by untargeted mass spectrometry-based methods as previously described (30, 37).

### Statistical Analysis

To evaluate differences in overall microbiota composition (i.e., β-diversity) between the groups, the analysis of similarities (ANOSIM) was performed on the weighted UniFrac distance matrixes. Shannon–Weaver and Chao1 diversity indices were calculated to assess the diversity of the GI microbiota using QIIME (38, 39). The indexes of bacterial diversity, sequencing data (percentage of species), qPCR data for each bacterial group, and metabolomics data were separately analyzed using a two-way repeated-measures analysis of variance (ANOVA) and tested to account for the effects of phase of treatment, time of measurement, and the interaction of phase and time. The normality of ANOVA residuals was evaluated *via* a Shapiro–Wilk *W* statistic, with the assumption of equal variances tested using the Levene’s *F* test. When necessary, the data were log, rank, or square root transformed as needed to meet the ANOVA normality assumption. The Phyla *Fusobacteria* and *Proteobacteria* required rank transformation, the Phylum *Bacteroidetes* required log transformation, while the remaining Phyla *Actinobacteria* and *Firmicutes* did not require transformation. Transformations for genera were performed as follows: log transformation for the genera *Streptococcus* and *Clostridium* and rank transformation for the genera *Bifidobacterium* and *Lactobacillus*. Adjustment for multiple comparisons was achieved *via* the Hochberg’s False Discovery Rate. A *p*-value of <0.05 was considered significant. Finally, as a pilot study, a power analysis was performed to determine the sample size needed to have an 80% chance of detecting overall differences between baseline (day 0) and day 30 of omeprazole administration for bacterial groups that had unadjusted *p*-values < 0.05 with an alpha values of 0.05. Commercially available software (SAS 9.4, SAS Institute Inc., Cary, NC, USA) was used for all statistical tests and the generation of summary statistics.

## Results

### Adverse Effects of Omeprazole Administration and Other Treatments

No cats developed adverse effects during the placebo phase of the study; however, one cat developed lower urinary tract signs. Although urinalysis and urine culture were unremarkable, antibiotic therapy was recommended by the overseeing laboratory veterinarian, and the cat received amoxicillin trihydrate/clavulanate potassium (Clavamox Drops, Zoetis Services LLC., Parsippany, NJ, USA) on day 10 to day 24 of placebo. Clinical adverse side effects were uncommon in the study cats. One cat developed intermittent hyporexia and diarrhea (fecal score 5) for 3 days while receiving omeprazole. The diarrhea quickly resolved with administration of metronidazole. Appetite and activity did not change before, during, and after antibiotic therapy for either cat that received antibiotics.

### Microbial Communities

Principal coordinate analysis plots did not yield any significant changes in weighted UniFrac distances with omeprazole administration (ANOSIM; *p* = 0.530). Omeprazole administration was also not associated with significant differences in the Shannon–Weaver or Chao1 indices of fecal bacterial diversity across the evaluated time points (*p* = 0.287 and *p* = 0.142, respectively).

**Figure 1:**
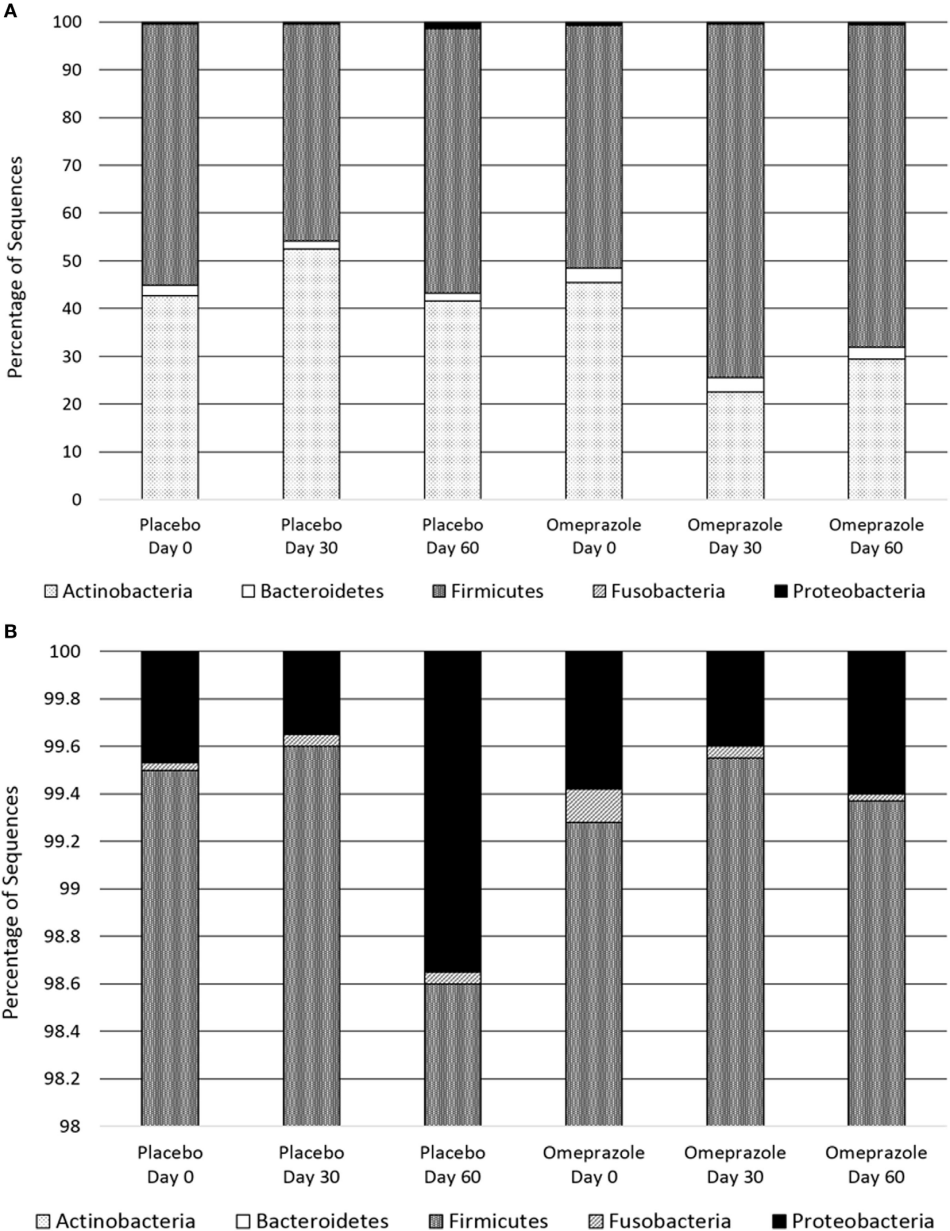
Composition of the fecal bacterial microbiota by phyla. **(A)** Data are presented for placebo and omeprazole at baseline (day 0), day 30, and day 60. The bars represent mean percentage of sequences, totaling 100% at each time point. **(B)** The same data, focused on Fusobacteria and Proteobacteria are presented for placebo and omeprazole at baseline (day 0), day 30, and day 60.

At baseline before either placebo or omeprazole administration, the most abundant phylum was Firmicutes followed by Actinobacteria, Bacteroidetes, Proteobacteria, and Fusobacteria (Figure 1). The sequence percentage of Proteobacteria at baseline (day 0) was significantly higher for omeprazole (T2, 0.6%) than for placebo (T1, 0.4%) (*p* = 0.014) (Table 1). No significant differences were noted in the sequence percentage of Firmicutes (*p* = 0.942), Actinobacteria (*p* = 0.619), Bacteroidetes (*p* = 0.936), and Fusobacteria (*p* = 0.783) in the omeprazole and placebo groups at baseline (Table 1).

**Table 1:** Relative abundances of bacterial phyla at baseline for placebo and omeprazole.

Phyla	Placebo	Omeprazole	*p*-Value
Firmicutes	54.592 (±28.63)	50.490 (±12.47)	0.9425
Actinobacteria	42.552 (±22.55)	45.418 (±13.83)	0.6191
Bacteroidetes	2.238 (±1.84)	2.967 (±4.40)	0.9361
Proteobacteria	0.436 (±0.33)	0.573 (±1.03)	0.0140
Fusobacteria	0.021 (±0.01)	0.013 (±0.27)	0.7833

*The data are presented as mean (±SD) percentage of each bacterial phyla*.

**Figure 2:**
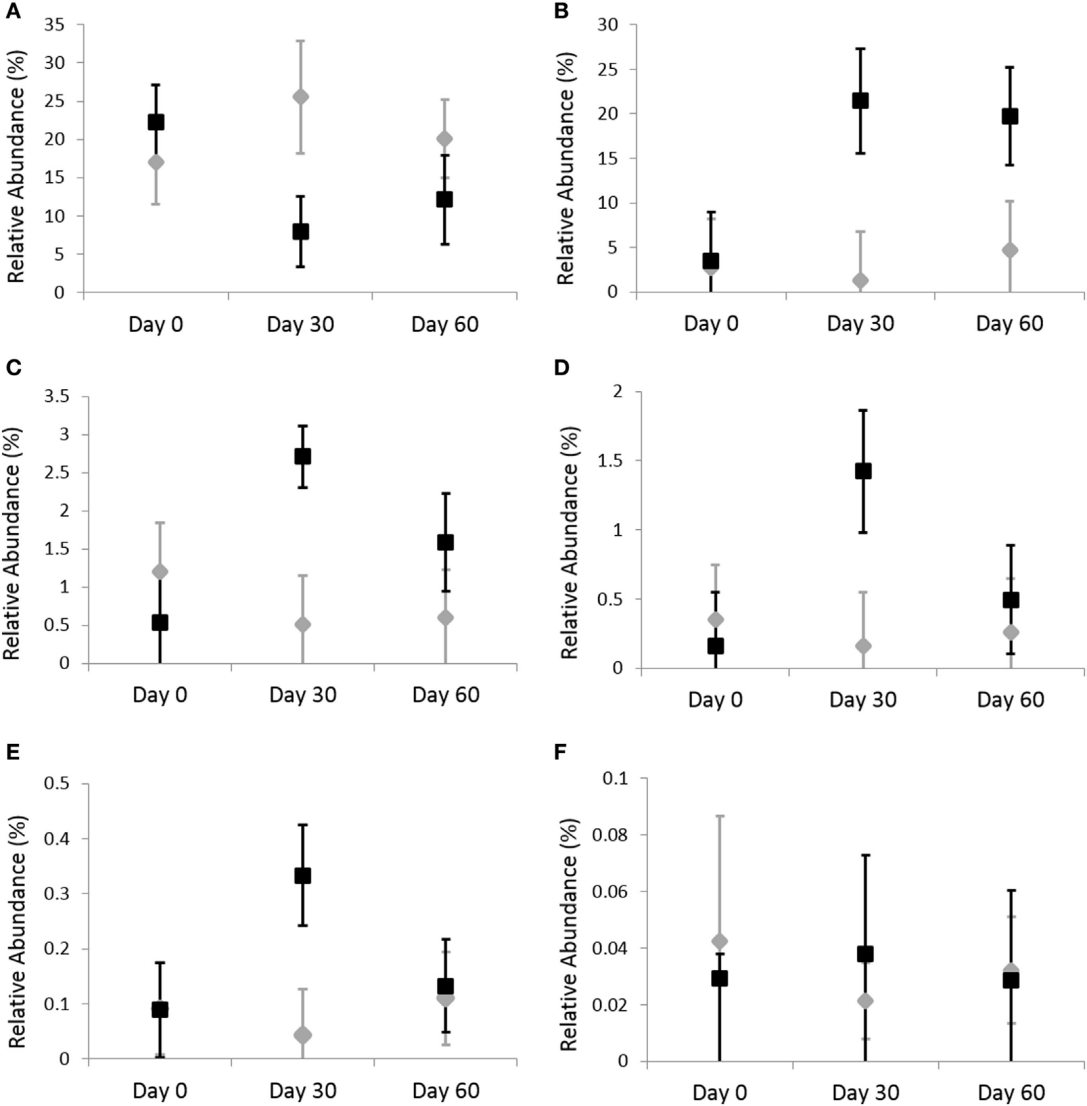
Relative abundances of fecal bacterial microbiota by genus. Data are presented for day 0 (baseline), day 30 (after 4 weeks of treatment), and day 60 (after 8 weeks of treatment) of placebo (250 mg lactose PO q 12 h, gray diamonds) and omeprazole (5 mg omeprazole PO q 12 h, black squares) administration. Error bars represent SEM. Notice that the *y*-axis is in a different scale for each bacterial group. **(A)** *Bifidobacterium*, **(B)** *Streptococcus*, **(C)** *Clostridium*, **(D)** *Lactobacillus*, **(E)** *Faecalibacterium*, and **(F)** *Helicobacter*.

**Figure 3:**
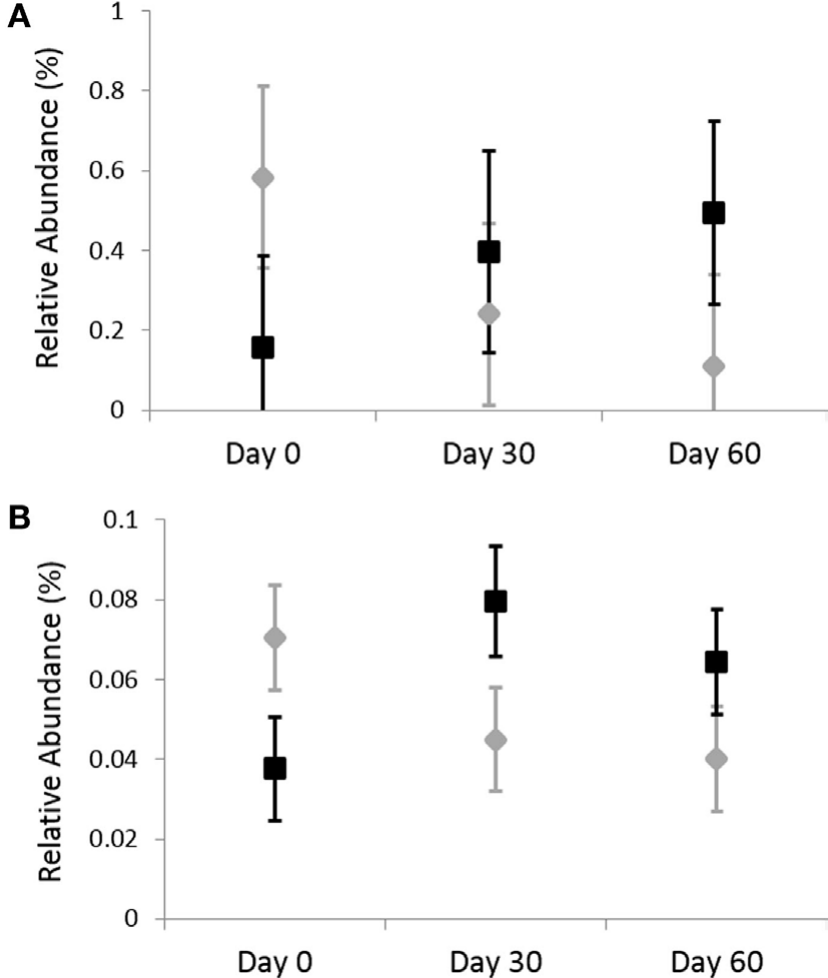
Relative abundances of fecal bacterial microbiota by species. Data are presented for day 0 (baseline), day 30 (after 4 weeks of treatment), and day 60 (after 8 weeks of treatment) of placebo (250 mg lactose PO q 12 h, gray diamonds) and omeprazole (5 mg omeprazole PO q 12 h, black squares) administration. Error bars represent SEM. Notice that the *y*-axis is in a different scale for each bacterial group. **(A)** *Clostridium perfringens* and **(B)** *Clostridium hiranonis*.

Omeprazole administration resulted in an increase in the sequence percentage of Firmicutes at 30 days (73.9%) and 60 days (67.4%) of omeprazole administration (unadjusted *p* = 0.023, Figure 1). This increase was driven by increases in the genera *Lactobacillus, Streptococcus, Clostridium*, and *Faecalibacterium* as shown in Figures 2B–E. The mean sequence percentage of *Lactobacillus* increased with omeprazole therapy, with the highest abundance compared with baseline (day 0, 0.2%) noted at day 30 (1.4%) of omeprazole therapy and a return toward baseline at day 60 (0.5%) of omeprazole therapy (unadjusted *p*-value = 0.009, Figure 2D). The mean sequence percentage of the genus *Streptococcus* was higher than baseline (3.5%) at 30 days (21.4%) and 60 days (19.8%) post-omeprazole therapy (unadjusted *p* = 0.024, Figure 2B). Compared with baseline (0.5%), the sequence percentage of *Clostridium* was also increased at 30 days (2.7%) and 60 days (1.6%) of omeprazole therapy (unadjusted *p* = 0.002, Figure 2C). The main species driving the increase in *Clostridium* were *C. perfringens* and *C. hiranonis* (Figure 3). Compared with baseline (0.09%), the sequence percentage of the genus *Faecalibacterium* was increased at 30 days (0.3%) of omeprazole therapy (unadjusted *p* = 0.040), returned to baseline at day 60 of omeprazole therapy (Figure 2E). However, significance was lost for the phylum Firmicutes and the genera *Lactobacillus, Streptococcus, Clostridium*, and *Faecalibacterium* that drove the change after adjustment for multiple comparisons (*p* = 0.080, *p* = 0.292, *p* = 0.363, *p* = 0.292, and *p* = 0.426, respectively).

Omeprazole administration resulted in a decrease from baseline (day 0, 45.4%) in the sequence percentage of Actinobacteria at 30 days (25.3%) and 60 days (29.5%) of omeprazole administration (unadjusted *p* = 0.007, Figure 1), but the difference among time points lost statistical significance with adjustment for multiple comparisons (*p* = 0.071). Within the phyla Actinobacteria, the only taxa that changed with omeprazole administration was the genus *Bifidobacterium*. Although not statistically significant after adjustment for multiple comparisons, the mean percentage of microbiota from the genus *Bifidobacterium* was lower (8.9%) at day 30 of omeprazole administration compared with all other time points (unadjusted *p* = 0.009, *p* = 0.292, Figure 2A). The sequence percentage of *Bifidobacterium* increased with continued omeprazole administration after day 30, but continued to be different from baseline (day 0) at day 60 of omeprazole treatment. However, significance was lost after multiple comparisons (*p* = 0.292).

Omeprazole administration was not associated with any significant alterations in the sequence percentages of the phyla Bacteroidetes, Proteobacteria, or Fusobacteria (*p* = 0.256, *p* = 0.071, *p* = 0.115, respectively). Within the phyla Proteobacteria, the sequence percentage of the genus *Helicobacter* was not affected by omeprazole administration (*p* = 0.531, Figure 2F).

### Quantitative Real-Time PCR

**Figure 4:**
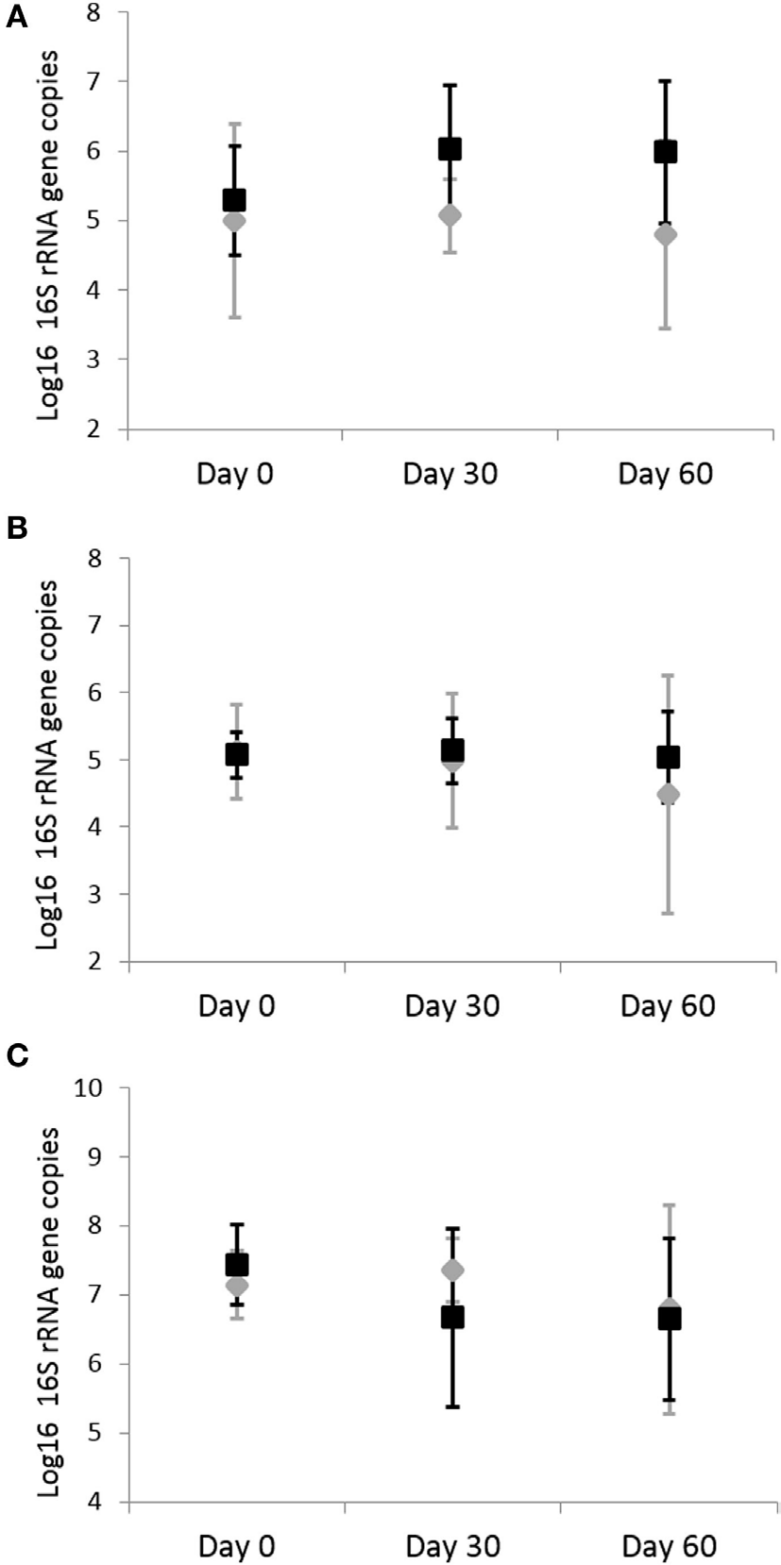
Quantitative real-time PCR results for fecal bacterial microbiota. Data are presented for day 0 (baseline), day 30 (after 4 weeks of treatment), and day 60 (after 8 weeks of treatment) of placebo (250 mg lactose PO q 12 h, gray diamonds) and omeprazole (5 mg omeprazole PO q 12 h, black squares) administration. Error bars represent SEM. Notice that the *y*-axis is in a different scale for each bacterial group. Notice that the *y*-axis is in a different scale for each bacterial group. **(A)** *Clostridium perfringens*, **(B)** *Clostridium hiranonis*, and **(C)** *Bifidobacterium*.

Analysis by qPCR revealed that there was no significant effects of omeprazole administration on the abundance of fecal *C. perfringens* (*p* = 0.703), *C*. *hiranonis* (*p* = 0.857), *C. difficile* (*p* = 0.703), and *Bifidobacterium* spp. (*p* = 0.438) (Figure 4). Before adjustment for multiple comparisons, only *Bifidobacterium* spp. was significant (unadjusted *p* = 0.038).

### Metabolomics

**Figure 5:**
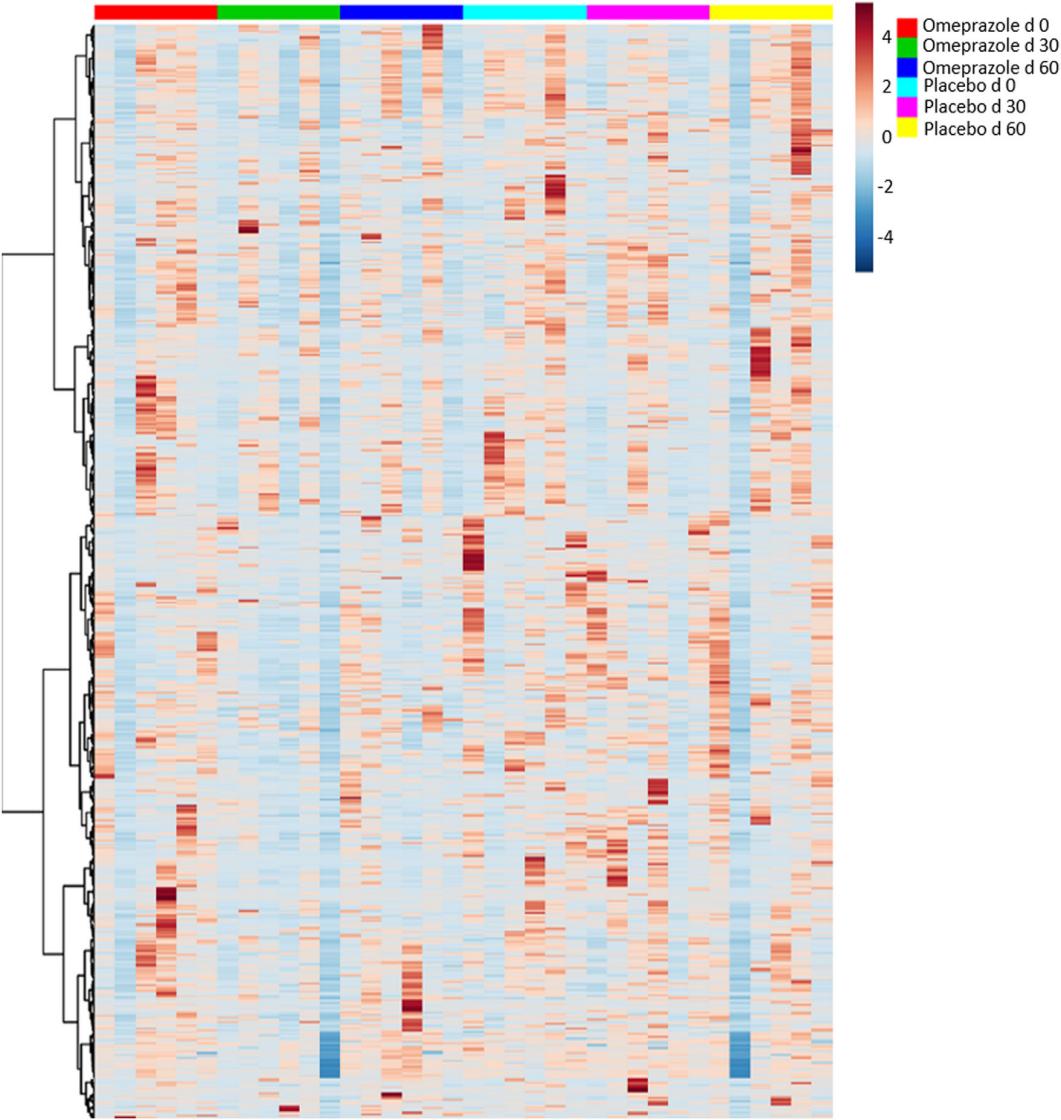
Heatmap showing relative distribution of metabolites. Each column represents an individual sample, and samples are grouped by day of treatment along the *x*-axis.

A total of 250 unique named and 428 unnamed compounds were identified in the feline fecal samples. After adjusting for multiple comparisons, none of these compounds changed significantly with the administration of omeprazole (Figure 5). However, unadjusted *p*-values showed that the fecal concentration of *O*-acetylserine and aminomalonate decreased with omeprazole therapy (unadjusted *p*-values 0.015 and 0.040, respectively, Table S1 in Supplementary Material). The concentration of ketohexose decreased at day 30 of omeprazole therapy (unadjusted *p* = 0.0387), but the concentration at day 60 was not different from baseline.

## Discussion

Proton pump inhibitors such as omeprazole are widely used in human and veterinary medicine for the treatment of acid-related disorders. Inhibition of gastric acid by PPI administration might result in an increased microbial load entering the small intestine. In people, PPI use has been associated with community-acquired pneumonia, *C. difficile-*associated disease, and bacterial and fungal overgrowth (8, 9, 24, 40–45). Omeprazole has been shown to alter the canine microbiome; however, no studies have explored its effect on the fecal microbiome and metabolome in cats despite its widespread use (13). The purpose of this pilot study was to explore the effect of prolonged omeprazole administration on the fecal bacterial microbiota and metabolome of healthy cats.

In this pilot study, oral omeprazole administration did not result in significant changes in the fecal microbiome and metabolome of healthy cats when administered for 60 days or less. Although there were no significant changes in this pilot study, our goal was to identify specific bacterial groups and their metabolites that warrant evaluation in a larger population of healthy cats and those with GI disease. Consequently, we report the relative abundance of several bacteria genera that changed with omeprazole administration, but lost significance with adjustment for multiple comparisons.

Even before omeprazole administration, the cats included in the current study had a high percentage of Actinobacteria due to a greater percentage of *Bifidobacterium* than what has previously been reported in the literature for healthy cats (46, 47). However, to date, these are the only two studies investigating the fecal microbiota of healthy cats.

Although significance was lost after adjustment for multiple comparisons, 16S rRNA sequencing results revealed that omeprazole administration increased the sequence percentage of *Streptococcus, Lactobacillus, Clostridium*, and *Faecalibacterium* spp. and decreased that of *Bifidobacterium* spp. in the feces of all cats. Predominating in the oropharynx, *Streptococcus* and *Lactobacillus* typically cannot survive in the acidic environment of the stomach. Consistent with previous studies in dogs, humans, and rats administered PPIs, the cats in this study had an increased sequence percentage of *Streptococcus* spp. and *Lactobacillus* spp. (12, 13, 48). This relative increase is likely a result of gastric colonization and proliferation of oropharyngeal bacteria secondary to PPI-induced acid suppression.

The sequence percentage of fecal *Clostridium* spp. increased with omeprazole administration in all cats, although the results were not significant after adjustment for multiple comparisons. *C. difficile*-associated disease is a major concern regarding the use of prolonged omeprazole treatment in people (8, 9, 42). The unadjusted 16S rRNA sequencing results from this pilot study in cats showed a relative increase in *C. hiranonis* and *C. perfringens*. Resulting in villus effacement, *C. perfringens* produces enterotoxins that stimulate mucosal fluid secretions and subsequent diarrhea. *C. hiranonis* results in the production of secondary bile acids that might damage the epithelium (49). *C. hiranonis* is thought to be a member of the normal large intestinal microflora; however, *C. hiranonis* was detected in duodenal tissue of 33.3% of dogs with GI disease compared with 16.7% of healthy dogs (50). A larger study population, ideally with collection of tissue samples, is indicated to evaluate the effect of prolonged omeprazole administration on the sequence percentage and persistence of changes in *Clostridium* spp.

Although significance was again lost after adjustment for multiple comparisons, omeprazole administration decreased the sequence percentage of fecal *Bifidobacterium* spp. in study cats. Cats with inflammatory bowel disease have a decrease in fecal *Bifidobacterium* compared with healthy cats (46). Therefore, Bifidobacteria might be helpful in promoting an anti-inflammatory environment, and decreases of Bifidobacteria might be harmful to cats at risk for primary GI disease. The decrease in fecal Bifidobacteria associated with omeprazole administration was transient, suggesting that the risk of prolonged omeprazole administration leading to altered *Bifidobacterium* abundance is likely low in healthy cats. However, more studies evaluating the effect of prolonged omeprazole administration on *Bifidobacterium* abundance in cats with IBD and other primary GI diseases are warranted.

Omeprazole administration increased the sequence percentage of *Streptococcus, Lactobacillus, Clostridium*, and *Faecalibacterium* spp. and decreased that of the *Bifidobacterium* spp. in the feces of all cats. These findings are consistent with that found in people, dogs, and rats administered PPI. However, because of the pilot study design and associated low sample size, statistical significance was lost after adjustment for multiple comparisons given the 50 statistical analyses required on the genus level alone. Therefore, further evaluation targeting these specific bacterial groups to limit the effects of multiple comparisons is indicated in a large population of cats, particularly cats with GI disease in which PPIs are commonly prescribed. According to the calculation of statistical power and sample size and assuming the same percentages of sequences, 45 cats would be necessary to achieve statistical significance in all of the bacterial groups for which the unadjusted *p*-value was <0.05.

The qPCR of fecal *C. perfringens, C. hiranonis, C. difficile*, and *Bifidobacterium* spp. failed to reveal any significant changes in these bacteria. Fecal samples are the most difficult specimens for DNA extraction and amplification as they often contain PCR inhibitors (51, 52). However, given the sequencing findings, the qPCR results further confirm that omeprazole does not cause significant alterations in the fecal microbiome of healthy cats.

One of the most novel aspects of this study is that it also included evaluation of the metabolome, which has yet to be extensively studied in humans that have received PPI. A total of 250 unique named and 428 unnamed compounds were identified in the feline fecal samples; however, after adjusting for multiple comparisons none of these compounds changed significantly with the administration of omeprazole. Before adjustment for multiple comparisons, the fecal concentration of *O*-acetylserine, aminomalonate, and ketohexose decreased with omeprazole therapy. *O*-acetylserine is a central metabolite of sulfur assimilation, providing the carbon backbone for synthesis of cysteine (53). Some sulfur-assimilating enteric bacteria, such as *Salmonella typhimurium* and *Klebsiella aerogenes*, are thought to utilize *O*-acetylserine or related compounds to sense the sulfur status of the gut environment (54, 55). Given their role in fermentation, the relative abundance of various enteric bacteria can also influence the concentrations of sugars such as the ketohexose identified in this study. As we begin to better understand the microbiome and its interactions with the metabolome, the impact of these metabolites on gut health in the cat will be better elucidated.

For all bacteria, except for *Streptococcus*, the unadjusted *p*-values only revealed a difference at day 30, with the sequence percentage approaching baseline at 60 days. This suggests that if omeprazole alters the fecal microbiome, the changes are likely mild and transient when given to healthy cats for 60 days or less. This conclusion is supported by the fact that no significant changes were seen in the fecal metabolome, which is largely influenced by the composition of the host GI microbiota.

In conclusion, results of this pilot study suggest that oral administration of omeprazole (0.83–1.6 mg/kg) to healthy cats has a mild impact on the fecal microbiome and metabolome when administrated for 60 days or less. Considering that the population of cats that receive omeprazole is more likely to be those with primary GI disease rather than healthy individuals, further investigation is warranted in a larger population of cats with GI disease. Based on the findings of this pilot study, the effect of omeprazole should be studied in a population of at least 45 cats. Furthermore, given the changes seen before adjustment for multiple comparisons, the bacterial groups that should be targeted include *Streptococcus, Lactobacillus, Clostridium, Faecalibacterium*, and *Bifidobacterium* spp. Evaluation of fecal metabolites such as *O*-acetylserine, aminomalonate, and ketohexose is also warranted.

## Ethics Statement

This study was carried out in accordance with the recommendations of the Institutional Animal Care and Use Committee at the University of Tennessee. The protocol was approved by the IACUC at the University of Tennessee (32312-0115).

## Author Contributions

SS participated in the conception and design of the study, sample collection, statistical analysis, and data interpretation and drafted the manuscript. MT participated in the conception and design of the study and sample collection and helped to draft the manuscript. JS participated in DNA extraction, microbiome and metabolome analysis, and study coordination and helped draft the manuscript. JP assisted in statistical analysis and helped to draft the manuscript.

## Conflict of Interest Statement

The authors declare no potential conflicts of interest with respect to the research, authorship, or publication of this article. Authors declare no off-label use of antimicrobials.

## Abbreviations

PPI, proton pump inhibitor; GI, gastrointestinal.
