# Supplementary material for: Omeprazole Minimally Alters the Fecal Microbial Community in Six Cats: A Pilot Study
Source: Front Vet Sci. 2018 Apr 16;5:79. doi: 10.3389/fvets.2018.00079 (PMC5911808; doi:10.3389/fvets.2018.00079)
Supplement: Supplementary file 1 [file Table_1.PDF]

Supplementary Table 1: Fecal concentrations of various metabolites with omeprazole administration. Data for each of the named metabolites are provided at each treatment time (day 0, day 30, and day 60) for placebo and omeprazole. The data are presented as median (range).

Metabolite	Placebo			Omeprazole			Unadjusted p-value
	0	30	60	0	30	60	
Zymosterol	3590 (202-12502)	3493 (138-5599)	4032 (110-8304)	3590 (202-12502)	1925 (663-7593)	5143 (2395-13212)	0.566
Xylulose NIST	4822 (2563-15795)	2973 (2145-8460)	5900 (4375-7939)	7088 (849-14870)	2813 (2042-4247)	4988 (2120-8777)	0.824
Xylose	192777 (60670-924367)	266901 (68734-503158)	368295 (154913-640112)	290089 (10311-637289)	102421 (77914-210649)	211761 (179366-380343)	0.436
Xylitol	2031 (790-3169)	1948 (1217-4828)	983 (610-4328)	839 (354-2521)	578 (115-1088)	1300 (468-2663)	0.052
Xanthine	17683 (6924-35260)	16295 (2789-33495)	17555 (8225-33075)	18264 (1630-34668)	14407 (5140-29697)	14771 (7891-61497)	0.834
Vanillic acid	1131 (98-3196)	1261 (117-3361)	485 (410-3057)	795 (152-2358)	542 (143-1245)	1050 (155-2891)	0.052
Valine	330150 (194192-449404)	251963 (35149-388225)	254851 (148144-788830)	228103 (30732-837778)	274762 (99021-406433)	232540 (123241-759320)	0.8276
Urocanic acid	3387 (116-6649)	3952 (295-6161)	3121 (587-4537)	3829 (625-7003)	1827 (935-7362)	2958 (1122-4622)	0.943
Uridine	3006 (1547-5188)	2199 (974-7278)	2748 (1192-13123)	2379 (802-6376)	1956 (837-8353)	2589 (423-5100)	0.855
Uric Acid	1222 (144-3765)	1062 (312-2777)	503 (123-1480)	1383 (80-2651)	2259 (324-5619)	1542 (420-6996)	0.242
Urea	1683 (570-445254)	9875 (566-237549)	5953 (1051-358269)	6949 (420-95749)	2107 (565-200213)	3817 (63-255942)	0.571
Uracil	53757 (4894-99090)	66326 (7310-149347)	50594 (18259-203678)	51342 (17223-81387)	42971 (22860-173015)	54239 (31764-214366)	0.814
UDP-glucuronic acid	1637 (656-5882)	1934 (375-3627)	1341 (801-2725)	1595 (711-3712)	1174 (77-2570)	1472 (117-3060)	0.318
Tyrosol	1932 (666-4636)	1359 (721-11067)	1922 (1642-22013)	1333 (434-8406)	1294 (164-2925)	1587 (488-3638)	0.462
Tyrosine	174954 (69487-310344)	187279 (40229-337298)	285581 (47576-742875)	140065 (10904-548162)	106396 (52126-297318)	153403 (45369-412185)	0.438
Tyramine	55919 (52406-1897594)	228176 (53528-847058)	185741 (82595-313777)	205967 (19932-396675)	129386 (43293-1143940)	315815 (54448-425243)	0.261
Tryptophan	69932 (3250-254002)	105606 (14802-534284)	138652 (3571-325379)	182647 (3153-451696)	51754 (15833-333644)	86002 (9009-293644)	0.247
Trehaloe	1234 (162-10427)	1837 (84-14409)	1619 (290-6526)	1533 (39-4734)	158 (132-575)	340 (76-1886)	0.159
Trans-4 hydroxyproline	7192 (1879-20825)	11225 (2019-51051)	11175 (2456-16176)	9242 (2529-33118)	3050 (892-9821)	5594 (2227-18442)	0.544
Tocopherol gamma	18854 (10540-34347)	10809 (1621-22549)	23187 (8677-52140)	15264 (79-34867)	12820 (97-23227)	16149 (69-46826)	0.887
Tocopherol delta-NOST	6009 (3284-9426)	2707 (952-7172)	7676 (3551-14779)	5746 (103-10107)	4291 (76-7013)	4243 (98-11843)	0.733
Tocopherol beta NIST	705 (362-1747)	339 (155-1235)	751 (625-2533)	458 (61-1787)	610 (51-847)	634 (27-1304)	0.553
Tocopherol alpha	583949 (371457-1077295)	347929 (97327-813921)	545901 (394098-1285398)	454670 (1419-1197354)	422545 (1749-766214)	362747 (2701-1076103)	0.663
Tocopherol acetate	191628 (16946-277076)	136875 (39360-332595)	274852 (115964-552651)	206374 (583-357307)	208666 (734-317018)	133469 (561-449725)	0.423
Thymine	20722 (8062-23850)	20546 (5366-32358)	15945 (11158-29855)	25477 (3274-42316)	20378 (5124-24093)	18435 (13982-56685)	0.620
Thymidine	4760 (2895-7973)	5979 (1699-9176)	4269 (3766-7788)	8478 (1233-14918)	6540 (1333-9905)	5779 (2446-11572)	0.632
Threonine	67919 (25772-99625)	56767 (13859-141197)	66175 (20101-128539)	77952 (4783-167223)	42731 (15087-50926)	52701 (37532-89791)	0.281
Threonic acid	709 (204-2074)	394 (286-2482)	782 (558-2531)	752 (87-1799)	417 (72-1235)	649 (403-2146)	0.904
Taurine	4906 (1561-24410)	19103 (3838-34127)	9322 (4532-88298)	7820 (1287-40810)	6215 (1784-10166)	7527 (943-41252)	0.276
Tagatose	4703 (2766-9779)	5363 (1314-6711)	5342 (2910-8965)	5486 (783-15662)	2252 (189-2709)	3112 (233-5983)	0.077
Sulfuric acid	476 (17-2374)	582 (81-3067)	1097 (55-3387)	702 (256-1996)	1153 (706-2426)	3186 (0-9844)	0.585
Sucrose	176 (68-431)	158 (103-696)	147 (76-201)	158 (56-203)	151 (99-333)	172 (140-245)	0.373
Succinic acid	**4478 (530-364077)**	**10049 (1220-269456)**	**2078 (706-37595)**	**4324 (923-28215)**	**776 (685-38372)**	**16041 (933-38580)**	**0.006**
Stigmasterol	10239 (8205-18662)	9876 (1294-19561)	8329 (3154-19830)	7933 (35-17890)	6921 (74-10467)	5048 (135-18354)	0.965

Steric acid	1037802 (601752-1461317)	541251 (440124-1103009)	1155128 (753297-1760831)	717742 (202534-1876485)	787595 (251528-1056548)	719008 (229717-1186671)	0.355
Squalene	8901 (2923-14697)	3613 (1661-14330)	12808 (6035-24736)	8962 (177-20169)	7477 (151-9552)	9747 (87-29172)	0.763
Sorbitol	1931 (368-8491)	1934 (389-2822)	2993 (1356-6107)	1783 (276-3322)	382 (195-1398)	1285 (618-1448)	0.132
Sinapinic acid	1657 (565-3225)	1788 (462-3597)	1893 (954-2640)	1671 (125-2170)	759 (189-1615)	948 (236-2544)	0.553
Serine	73038 (26855-97408)	60784 (7673-148760)	73596 (24034-155361)	71448 (11296-225520)	41392 (20132-68152)	66442 (30840-82192)	0.525
Saccharopine	259 (127-974)	316 (147-501)	521 (252-1470)	401 (56-1111)	185 (95-1026)	425 (102-1937)	0.838
Ribose	121165 (41031-207791)	112734 (37279-207044)	138447 (59534-262098)	131798 (32322-283041)	95042 (63636-128810)	119771 (55038-239071)	0.457
Ribonic acid	465 (196-1677)	636 (180-2142)	737 (258-1944)	410 (85-896)	373 (201-624)	560 (368-939)	0.447
Raffinose	187 (75-1430)	2254 (47-1220)	135 (48-597)	253 (24-404)	43 (25-85)	129 (37-237)	0.578
Quinolinic acid	278 (42-1721)	414 (46-1428)	507 (20-2026)	481 (31-1037)	533 (66-977)	762 (11-1250)	0.344
Pyruvic acid	929 (471-1704)	829 (190-1114)	1308 (878-1405)	743 (344-1199)	273 (87-1189)	1025 (348-1701)	0.728
Putrescine	215382 (8192-858939)	236166 (13001-1004909)	407229 (72346-2615671)	708076 (33622-1522178)	759038 (3401-1522197)	115569 (15325-1889998)	0.476
Pseudo urindine	26734 (12652-35423)	14842 (8299-26589)	19812 (15996-37771)	22411 (854-33238)	16575 (2047-32841)	17362 (8700-34422)	0.649
Propane-1,3,diol NIST	735 (103-3678)	671 (318-1436)	469 (313-840)	393 (253-857)	395 (336-1530)	665 (266-1080)	0.244
Proline	121642 (13612-213631)	97307 (29155-159849)	69486 (51508-336302)	141768 (24235-434680)	107568 (3640-200987)	95955 (21366-309010)	0.291
Piperidone	34886 (20037-84020)	63222 (8822-163591)	75816 (26612-365281)	62436 (26194-89625)	51807 (7025-238954)	25243 (1714-113505)	**0.021**
Pipecolinic acid	1244 (533-6642)	3178 (639-4643)	3114 (1364-4211)	2209 (844-5080)	2301 (318-4526)	1104 (309-4050)	0.283
Pinitol	1376 (117-18139)	2447 (136-4744)	191 (115-8158)	323 (135-1900)	95 (71-310)	451 (132-3450)	0.052
Pimelic acid	428 (215-1250)	743 (206-1183)	760 (293-1125)	595 (293-989)	755 (192-983)	749 (283-938)	0.657
P-hydroxylphenyllactic	583 (333-3354)	1247 (287-2916)	796 (428-1899)	557 (81-2273)	175 (150-1125)	555 (168-2235)	0.342
Phosphoethanolamine	277 (24-441)	186 (36-588)	113 (46-2056)	106 (3-1099)	63 (45-187)	207 (46-1097)	0.726
Phosphate	5117 (1798-11065)	4161 (496-6858)	6721 (3132-8154)	3514 (1033-11594)	5366 (1131-6171)	5839 (2801-6834)	0.755
Phenylethylamine	5225 (472-54536)	1291 (527-11551)	1545 (834-18067)	1147 (256-4905)	1086 (214-6637)	731 (451-5600)	0.409
Phenylalanine	132705 (42709-266351)	96326 (18959-226037)	130969 (86931-309357)	88591 (7788-242657)	77430 (26368-129765)	74331 (36342-243487)	0.536
Phenylacetic acid	27887 (4206-78946)	18937 (9828-59054)	47089 (16677-83762)	28068 (11285-42750)	45881 (3006-52410)	24464 (2941-58931)	0.296
Phenaceturic acid	9501 (1939-31098)	7606 (1256-18020)	13530 (4515-23414)	7005 (1670-14355)	2706 (1346-22533)	6833 (2743-57727)	0.962
Pentadeconic acid	50846 (12219-196839)	35748 (6069-11794)	87072 (31693-10543)	33078 (1701-245512)	44303 (2341-89116)	33799 (3032-98593)	0.693
Pelargonic acid	6652 (6517-8961)	6944 (6557-7732)	6991 (4238-7906)	6059 (4944-8572)	6048 (5447-6352)	6118 (1422-9981)	0.510
Parabanic acid NIST	1163 (862-2191)	2955 (2276-3522)	2105 (1433-3215)	1738 (835-3100)	2884 (1256-3333)	3158 (1335-3959)	0.157
Pantothenic acid	**1785 (228-2762)**	**1303 (436-3493)**	**5068 (2175-7396)**	**2649 (226-3255)**	**1999 (316-3098)**	**1392 (776-5067)**	**0.022**
Palmitoleic acid	3085 (1221-5253)	1792 (440-2689)	2557 (1421-6074)	2139 (25-4949)	1698 (59-9498)	2080 (62-4062)	0.633
Palmitic acid	159840 (74442-204373)	77329 (57165-144155)	146363 (105478-233063)	100786 (2238-340900)	97737 (33716-180603)	100224 (29824-169683)	0.419
Oxoproline	94633 (28564-13114)	124807 (52725-252635)	97090 (66415-187333)	132574 (21796-158392)	87814 (1250-168371)	90040 (74728-331798)	0.186
Oxalic acid	128 (64-1861)	81 (19-350)	369 (56-615)	62 (12-319)	163 (82-216)	256 (25-587)	0.864
Orotic acid	4251 (1134-11056)	3084 (1318-7541)	2276 (1056-10572)	4386 (518-11742)	3552 (342-3625)	2841 (2207-9102)	0.864
Ornithine	34545 (8944-79079)	26559 (9077-95721)	53715 (33497-114212)	58944 (7998-139028)	54221 (2005-127696)	36360 (3318-66102)	0.202
Oleic acid	65744 (38024-90684)	32757 (899-65381)	74538 (35098-130571)	57712 (396-112391)	30312 (288-112551)	44494 (378-68021)	0.263

Oleamide NIST	4875<br>(1940-15263)	11165<br>(2773-32173)	5669<br>(3667-10602)	4187<br>(94-7793)	3285<br>(735-10514)	3078<br>(744-8978)	0.629
Octadecylglycerol	16028<br>(644-49388)	6063<br>(1643-54329)	17678<br>(8938-48714)	9953<br>(514-84757)	15957<br>(92-27720)	11325<br>(1648-39277)	0.736
Octadecanol	2289<br>(694-10266)	945<br>(272-5665)	2504<br>(1213-7123)	1182<br>(248-10989)	1121<br>(329-3767)	2539<br>(178-5664)	0.789
O-acetylserine	492<br>(156-1272)	1132<br>(488-1502)	811<br>(547-1740)	729<br>(126-2196)	473<br>(114-1262)	269<br>(70-358)	**0.015**
Norvaline	8785<br>(1296-20900)	2892<br>(1030-8016)	3339<br>(710-1558)	2878<br>(189-5441)	758<br>(281-91778)	2731<br>(1041-4634)	0.529
Nonadecanoic acid	3949<br>(2595-7063)	3095<br>(2331-4581)	5121<br>(3243-7678)	3097<br>(185-6943)	3572<br>(357-4217)	3255<br>(674-4482)	0.474
N-methylalanine	78326<br>(35646-124123)	28608<br>(4514-104795)	72609<br>(25688-116436)	40183<br>(2620-69417)	54472<br>(4408-72557)	69513<br>(3975-164159)	0.143
Nicotinic acid	21527<br>(6905-32626)	19997<br>(8840-38905)	23976<br>(20012-61927)	23383<br>(3964-38865)	12778<br>(5217-46328)	22802<br>(7324-35984)	0.505
N-acetylputrescine	1767<br>(1439-2366)	2968<br>(1147-15342)	3389<br>(907-6018)	6451<br>(2600-20345)	30484<br>(1493-57964)	4064<br>(540-8782)	0.179
N-acetylornithine	1615<br>(917-2977)	2157<br>(915-4499)	2852<br>(1619-5286)	2086<br>(901-3067)	1136<br>(375-4329)	1789<br>(443-3699)	0.201
N-acetylglutamate	350<br>(302-804)	652<br>(222-1258)	495<br>(263-1294)	369<br>(236-1242)	440<br>(216-746)	826<br>(101-1494)	0.300
N-acetyl-D-mannosamine	7934<br>(3860-27892)	9460<br>(3559-25734(	7041<br>(5727-13638)	12332<br>(1465-33943)	4027<br>(1748-4953)	7722<br>(898-9526)	0.121
N-acetyl-d-hexosamine	2098<br>(443-2594)	1838<br>(443-2594)	2280<br>(714-3843)	2320<br>(316-3054)	738<br>(431-1807)	1414<br>(993-2362)	0.159
N-acetyl-D-galactosamine	15323<br>(7830-42650)	17679<br>(8636-40228)	14782<br>(10187-51312)	21014<br>(5333-84337)	9122<br>(3351-14059)	11552<br>(440-21189)	0.159
N-acetylaspartic acid	1680<br>(455-8265)	2836<br>(340-5224)	4046<br>(1212-5225)	4340<br>(323-6794)	1486<br>(190-3826)	2881<br>(782-4862)	0.526
Myristic acid	3915<br>(1522-10195)	2702<br>(1336-5393)	3863<br>(2741-11012)	2935<br>(461-14794)	4649<br>(782-11638)	3776<br>(518-4930)	0.235
Myo-inositol	3783<br>(596-13674)	4311<br>(987-10137)	3341<br>(2040-8409)	3332<br>(917-6059)	2575<br>(729-4983)	2683<br>(1001-9642)	0.608
Methionine sulfoxide	77515<br>(26285-128156)	57058<br>(18246-234821)	79409<br>(23703-187275)	79455<br>(3622-182580)	56749<br>(17340-154462)	60810<br>(12380-95980)	0.805
Methionine	4941<br>(1184-12825)	2546<br>(633-11203)	3144<br>(1337-9764)	3413<br>(30-39642)	3658<br>(1034-7031)	2621<br>(1771-31665)	0.810
Mannose	6957<br>(5133-22095)	5522<br>(1861-21177)	10111<br>(5593-10961)	11701<br>(8055-66722)	5333<br>(3655-9327)	8179<br>(5133-22095)	0.205
Maltotriose	368<br>(81-1754)	284<br>(66-1380)	256<br>(246-629)	357<br>(61-1542)	180<br>(27-208)	177<br>(39-1021)	0.094
Maltose	14503<br>(2184-230278)	23709<br>(903-112365)	10148<br>(5066-73504)	24779<br>(431-54588)	2794<br>(582-8508)	4422<br>(354-57757)	0.164
Malonic acid	280<br>(160-706)	200<br>(142-287)	484<br>(294-754)	261<br>(78-560)	403<br>(58-632)	360<br>(101-590)	0.317
Malic acid	553<br>(387-1169)	356<br>(287-974)	362<br>(242-1434)	828<br>(41-1243)	456<br>(103-869)	726<br>(156-4442)	0.697
Maleimide	1745<br>(1164-5044)	2359<br>(936-4123)	3125<br>(622-4984)	1375<br>(4456-6196)	1100<br>(372-4998)	1747<br>(660-2697)	0.287
Lyxose	40757<br>(19575-72632)	74227<br>(21932-157062)	50134<br>(132733-205219)	82762<br>(612-228976)	5603<br>(4109-59650)	58375<br>(14481-125385)	**0.028**
Lyxitol	4421<br>(1248-10075)	3999<br>(1065-6369)	332<br>(2318-10815)	3508<br>(528-6239)	1815<br>(367-3667)	2188<br>(1650-8757)	0.837
Lysine	102895<br>(20382-207534)	68319<br>(18523-187383)	177799<br>(29906-247003)	126319<br>(2785-427724)	136924<br>(8221-334889)	87324<br>(3876-220920)	0.403
Lithocholic acid	10654<br>(2949-130830)	7896<br>(1669-47762)	12084<br>(1431-108114)	7661<br>(115-69612)	35568<br>(474-71957)	7020<br>(58-55261)	0.155
Linolenic acid	23032<br>(3010-59071)	13271<br>(5895-47378)	16275<br>(6514-98279)	19219<br>(200-32558)	18714<br>(182-51147)	15207<br>(160-47909)	0.394
Linoleic acid	21878<br>(14036-59952)	12571<br>(5291-50257)	34800<br>(5306-98759)	25596<br>(249-33932)	19286<br>(259-54682)	25168<br>(215-44873)	0.333
Lignoceric acid	3211<br>(2448-7880)	2203<br>(1081-4337)	6320<br>(2485-10449)	3676<br>(106-9938)	1780<br>(212-5229)	2710<br>(220-5516)	0.115
Leucine	356420<br>(143169-448366)	223244<br>(33833-386527)	170647<br>(113460-672090)	195395<br>(20619-796792)	265522<br>(100786-384002)	183172<br>(95120-512755)	0.707
Lanosterol	2151<br>(1549-8388)	1397<br>(604-2923)	2305<br>(1484-9410)	3052<br>(44-4718)	1516<br>(41-4102)	1369<br>(77-4550)	0.539
Lactose	282<br>(116-1930)	237<br>(40-1046)	883<br>(127-2429)	229<br>(27-1010)	112<br>(56-169)	190<br>(87-426)	0.712
Lactitol	1074<br>(17-3499)	832<br>(237-2517)	495<br>(61-1683)	590<br>(166-2631)	82<br>(31-500)	268<br>(23-2012)	0.211
Lactic acid	5224<br>(3453-687807)	9358<br>(2408-60498)	15488<br>(1457-173410)	13952<br>(1275-36267)	3281<br>(1808-93531)	7504<br>(3489-51670)	0.567
Ketohexose	181<br>(87-1013)	230<br>(73-737)	302<br>(204-576)	288<br>(84-544)	90*<br>(68-129)	233<br>(57-398)	**0.0387**

Isothreonic acid	1354 (279-3073)	1408 (313-3381)	1209 (538-3503)	880 (114-2861)	1141 (117-1501)	1146 (633-2370)	0.893
Isothreitol	250 (29-4044)	874 (20-4165)	546 (260-2939)	353 (64-2126)	116 (70-474)	421 (102-1748)	0.290
Isoribose	1156 (505-2475)	1273 (245-2843)	1611 (960-13194)	1362 (234-17570)	508 (60-1186)	1276 (464-8892)	0.185
Isopentadecanoic acid	94343 (2055-321930)	55429 (8783-235985)	135555 (62583-202890)	57143 (2697-362207)	73801 (3617-138558)	73914 (7374-173455)	0.739
Isomaltose	677 (129-7220)	908 (360-5319)	551 (381-1617)	1039 (135-189377)	229* (172-427)	357 (235-1071)	**0.017**
Isoleucine	261259 (128256-380704)	196645 (25570-432947)	213448 (85194-634612)	179529 (21456-650015)	205033 (64813-277240)	186302 (98947-638990)	0.881
Isoheptadecanoic acid NIST	4180 (700-21210)	2220 (1057-9219)	4369 (1662-10117)	1515 (143-14025)	3202 (70-4729)	2158 (1076-9871)	0.879
Inositol-4-monophosphate	402 (192-3055)	355 (104-2495)	1347 (448-2375)	373 (55-641)	365 (30-803)	715 (57-1967)	0.799
Inosine	251 (62-414)	258 (125-338)	277 (41-328)	196 (93-620)	168 (106-2224)	266 (42-743)	0.981
Indole-3-acetate	23639 (5370-55962)	19123 (5370-55692)	29585 (14362-42317)	19731 (5158-37486)	15806 (3226-26178)	17954 (2496-29269)	0.433
Hypoxanthine	27956 (17307-63983)	33319 (10082-50614)	35721 (13045-58974)	27995 (890-74725)	31225 (20736-51161)	38515 (21340-97238)	0.683
Hydroxylamine	5280 (4411-17765)	8297 (2456-31894)	9459 (2696-13395)	7413 (4940-16945)	7719 (4274-16182)	12375 (4264-37074)	0.541
Hydroquinone	479 (180-3415)	236 (165-991)	547 (190-1834)	757 (46-2579)	316 (72-483)	616 (165-3418)	0.696
Hydrocinnamic acid	8620 (1489-289243)	3123 (454-585362)	48048 (2058-125563)	18055 (717-71955)	80349 (1592-267396)	30984 (402-157899)	0.160
Homoserine	1556 (832-2013)	1214 (361-1880)	1455 (727-2377)	937 (165-2661)	319 (159-484)	696 (168-2496)	0.141
Hexuronic acid	4811 (1556-15462)	8316 (1743-10647)	8127 (4930-8739)	5719 (299-6896)	3279 (2064-5336)	4961 (2518-1121)	0.716
Hexose	239 (35-863)	273 (105-6888)	737 (73-6668)	380 (275-596)	158 (23-379)	144 (65-779)	0.103
Hexadecylglycerol NIST	4719 (1652-8053)	2471 (991-9884)	5596 (2190-13476)	3127 (78-7230)	3538 (62-7110)	2736 (117-6128)	0.217
Heptadecanoic acid	18673 (6973-57798)	10450 (5440-24532)	21698 (12844-36783)	10849 (5354-49369)	17965 (1559-21470)	14047 (1480-29827)	0.671
Guanosine	134 (61-506)	162 (90-297)	145 (65-293)	129 (66-508)	100 (38-1456)	91 (56-557)	0.650
Guanine	2920 (502-7574)	2198 (795-5706)	3483 (987-7427)	2207 (243-4119)	2610 (433-3516)	2993 (896-4791)	0.500
Glycyl-proline	16497 (2139-40328)	4888 (912-15650)	10795 (1600-33426)	9645 (1590-24621)	2859 (978-19079)	7530 (914-38722)	0.229
Glycine	72562 (19592-125705)	81951 (16724-181638)	78229 (16168-112341)	77385 (9761-137226)	48075 (13006-66445)	65605 (16844-271465)	0.254
Glycerol-alpha-phosphate	1273 (801-1750)	1677 (584-2838)	1501 (631-4491)	1130 (138-5106)	1116 (116-2803)	1129 (199-3760)	0.537
Glycerol-3-galactoside	2173 (672-13925)	1994 (839-3645)	1575 (820-3576)	1653 (170-4223)	820 (221-1241)	673 (4506-2898)	0.422
Glycerol	46028 (21471-75896)	24824 (11619-49785)	44842 (19643-73635)	28477 (18490-93906)	26551 (18402-28952)	40560 (12125-99619)	0.764
Glyceric acid	5252 (1068-12400)	6291 (1897-14084)	7644 (1325-20191)	6015 (822-12195)	4941 (3831-11236)	7144 (3844-9375)	0.589
Glutaric acid	837 (269-1535)	867 (144-1776)	849 (593-2166)	666 (211-1755)	454 (238-2963)	482 (79-1924)	0.868
Glutamine	3039 (590-19612)	2089 (366-39592)	6185 (394-31521)	9083 (120-24044)	2910 (433-14328)	3312 (93-12519)	0.271
Glutamic acid	148168 (34494-196521)	104288 (55255-229312)	159441 (93119-322864)	182439 (42272-250450)	174499 (80154-209853)	172959 (92087-408239)	0.986
Glucose-1-phosphate	1111 (401-1925)	1722 (419-2435)	690 (514-1096	856 (408-2893)	522 (148-1274)	631 (446-1451)	0.196
Glucose	86714 (24979-382150)	65109 (16442-391102)	83341 (20700-100799)	99875 (7922-356383)	39915 (19122-42927)	35596 (2908-136875)	0.255
Glucoheptulose	1392 (625-2284)	683 (306-2461)	872 (544-1941)	697 (361-1100)	270 (174-1092)	580 (170-1530)	0.854
Galacturonic acid	3026 (1635-12653)	2023 (788-16634)	4533 (1662-17892)	3411 (298-15860)	1549 (1340-7192)	3563 (988-8105)	0.780
Galactose	332988 (54329-488141)	116490 (45580-344960)	411705 (96344-458036)	166888 (40551-554662)	205801 (92874-232240)	178549 (8693-566119)	0.091
Galactinol	359 (37-1029)	589 (244-2119)	624 (33-1218)	458 (38-5141)	196 (35-633)	211 (81-468)	0.132
Fumaric acid	1856 (1265-2727)	2154 (1217-2833)	2234 (1899-2464)	1842 (413-6520)	1355 (292-2011)	2270 (1473-4180)	0.065
Fucose	66524 (27688-305474)	133622 (24729-239011)	96034 (18704-204054)	63999 (16512-348961)	39002 (13678-49396)	59844 (11418-227870)	0.204

Fructose	2632 (1476-5739)	3017 (891-4257)	3072 (1825-5494)	2612 (346-10129)	1310 (97-2080)	1603 (34-3553)	0.084
Ferulic acid	1991 (561-5426)	1953 (459-3303)	1601 (974-5574)	1917 (100-3604)	985 (447-1256)	995 (216-1626)	0.312
Ethanolamine	21947 (10315-32324)	24060 (12462-27405)	34522 (10695-38455)	23179 (10882-67863)	18620 (12382-26943)	18625 (1687-46792)	0.098
Erythritol	3093 (174-21333)	4720 (356-17152)	2092 (300-11965)	2536 (470-7035)	528 (152-4058)	3639 (252-12924)	0.140
Enolpyruvate NIST	256 (152-323)	276 (223-338)	271 (219-506)	309 (110-361)	197 (153-425)	292 (208-415)	0.306
Dopamine NIST	42678 (1066-99734)	29661 (3850-563541)	54461 (2180-467258)	73359 (1071-288234)	32995 (950-184747)	13526 (408-186243)	0.372
Dodecanol	302 (206-625)	298 (215-418)	355 (238-1637)	310 (212-413)	333 (254-377)	325 (190-1752)	0.476
Docosenoic acid	3998 (1512-7500)	2985 (1732-7030)	3190 (1892-6923)	2338 (140-47019)	1753 (172-4458)	2217 (835-5545)	0.728
Docosahexaenoic acid	4642 (337-13518)	1626 (163-7160)	4232 (549-16907)	1938 (115-9403)	7775 (87-15811)	1240 (101-6684)	0.083
Dihydroxyacetone	1338 (705-7611)	1638 (613-6277)	1621 (487-8146)	1385 (400-1846)	1295 (670-4589)	1576 (672-5991)	0.968
Dihydrocholesterol	8688 (1789-20086)	3880 (1246-12429)	10643 (4016-22322)	7698 (43-18997)	6438 (143-9385)	4749 (63-17343)	0.673
Diglycerol	4362 (2364-9113)	4107 (1943-7984)	3088 (1146-11968)	4711 (1061-13871)	3280 (613-8087)	2239 (1517-8784)	0.248
Digalacturonic acid	343 (219-3457)	489 (109-1294)	453 (151-577)	450 (42-592)	147 (53-355)	273 (26-338)	0.570
d-erythro-sphingosine	16575 (8737-41872)	13058 (4200-37021)	30343 (20532-102698)	19995 (6-145653)	13284 (143-37006)	15114 (169-65148)	0.303
Deoxycholic acid	362387 (51220-892925)	100738 (12430-292678)	349042 (39943-900130)	162211 (804-278333)	234717 (8863-538388)	83691 (5827-625243)	0.070
Dehydroabietic acid	1140 (809-1454)	1021 (671-1522)	1145 (849-1642)	870 (578-1366)	910 (630-1237)	1002 (647-1538)	0.878
Daidzein	497 (42-1007)	365 (59-549)	687 (96-942)	321 (61-685)	99 (18-277)	156 (46-351)	0.524
Cytosin	494 (291-1813)	645 (378-1101)	562 (460-1351)	684 (226-1406)	489 (246-2717)	554 (358-1261)	0.874
Cysteine	3773 (1020-5295)	3354 (929-5509)	3109 (1797-6635)	2731 (82-8473)	1069 (541-6035)	1588 (1001-3513)	0.817
Creatinine	5457 (1410-168487)	9677 (3490-154531)	7177 (1378-30191)	10057 (674-130400)	3031 (323-336419)	11233 (1177-98033)	0.598
Citrulline	12213 (3336-19921)	12327 (4731-18479)	22921 (5566-28592)	18319 (1416-46636)	14106 (1945-29566)	14209 (6760-38847)	0.587
Citric acid	240 (130-434)	201 (67-992)	222 (82-269)	139 (58-416)	346 (54-577)	332 (152-972)	0.136
Citramalic acid	357 (256-508)	430 (279-1150)	437 (246-1341)	296 (108-1066)	239 (68-448)	360 (136-626)	0.115
Cis-sinapinic acid	173 (48-436)	271 (53-1196)	90 (78-305)	155 (23-549)	122 (35-228)	208 (36-957)	**0.049**
Cis-gondoic acid	14084 (7664-28397)	4962 (2735-10853)	15290 (13798-24432)	10063 (455-58682)	14659 (355-21151)	7566* (240-14518)	**0.0352**
Cholic acid	55694 (5897-111274)	99138 (1059-224218)	26727 (1196-164342)	16047 (554-139109)	7390 (203-126580)	19774 (623-65070)	0.930
Cholesterol	450717 (148444-694820)	238279 (97562-682848)	380812 (298672-935137)	369373 (7328-999673)	57235 (1034-574904)	308825 (2543-820827)	0.201
Chenodeoxycholic acid	2739 (1176-16468)	2712 (1032-49034)	4526 (1771-7979)	2994 (517-47704)	1349 (261-63357)	1817 (534-12856)	0.394
Cerotinic acid	606 (364-998)	554 (239-1085)	1007 (355-3390)	647 (140-2429)	1097 (127-1551)	823 (161-1416)	0.157
Cellobiose	12318 (3663-65422)	15666 (1279-39585)	5623 (3241-61159)	15803 (388-58856)	2575 (717-23173)	19543 (434-27599)	0.231
Capric acid	688 (553-820)	559 (482-779)	676 (569-1328)	633 (377-729)	542 (440-1070)	712 (356-1363)	0.513
Butyrolactam NIST	1702 (1270-3143)	1948 (1194-2193)	2278 (1616-9855)	2038 (639-3355)	3059 (1076-3726)	2166 (1367-4913)	0.230
Butane-2,3-diol NIST	10164 (4753-18904)	10802 (3266-24224)	17410 (5393-36969)	13716 (2728-29096)	8733 (2387-22014)	20988 (9814-59549)	0.494
Biphenyl	1052 (226-5530)	2069 (92-6969)	1075 (479-14068)	1424 (459-2209)	753 (111-2188)	534* (47-1391)	**0.026**
Beta-sitosterol	676459 (399004-799700)	393434 (126385-710305)	644320 (539669-963501)	515167 (2369-951753)	426994 (1041-714382)	471305 (2964-935297)	0.726
Beta-glycerolphosphate	340 (140-702)	558 (90-858)	441 (163-682)	323 (158-620)	139 (72-807)	204 (119-755)	0.305
Beta-glutamic acid	1585 (295-9307)	2652 (181-5654)	1298 (215-3197)	1296 (92-2301)	370 (29-1306)	1348 (73-12246)	0.247
Beta-gentiobiose	1584	1479	1408	1377	703	825	0.663

	(378-5673)	(564-3054)	(587-3267)	(88-20275)	(120-1025)	(119-2395)	
Beta-alanine	2986 (2005-13281)	5206 (756-16488)	6451 (4320-68126)	4588 (2224-15038)	4289 (747-33050)	3430 (1193-10988)	0.194
Benzoic acid	3393 (2350-4692)	3042 (1858-5706)	3749 (3065-4982)	3211 (1454-4401)	2579 (2203-2767)	3457 (1785-23851)	0.851
Behenic acid	16489 (7197-35000)	7693 (6057-36171)	15411 (13487-33305)	12090 (1389-47435)	15010 (1077-23184)	11902 (870-21686)	0.641
Aspartic acid	36925 (11298-44536)	31211 (13825-107263)	31942 (19325-150168)	43478 (11831-67237)	31597 (24344-102298)	77440 (19081-335928)	0.895
Asparagine	1716 (657-11298)	1575 (1143-12222)	1568 (1365-3596)	1277 (251-9157)	977 (646-4582)	1597 (667-5209)	0.732
Arachidonic acid	24608 (6327-60330)	9080 (2732-31431)	16759 (6825-79895)	11267 (109-31032)	18878 (68-35613)	10243 (96-41134)	0.447
Arachidic acid	20835 (10854-38267)	11470 (9410-26242)	25660 (13944-40459)	14744 (2060-43521)	13360 (3167-21318)	15952 (2132-25130)	0.415
Arabitol	4275 (1248-9983)	4022 (1065-5935)	3332 (2318-9918)	2375 (528-6239)	1815 (412-3667)	2230 (1650-8161)	0.986
Aminomalonate	2363 (383-4081)	3292 (432-4391)	1255 (476-4443)	2861 (148-5171)	1366 (314-3610)	1192* (680-2043)	**0.040**
Alpha-aminoadipic acid	636 (169-1291)	402 (170-1084)	1046 (275-1194)	459 (134-1259)	576 (76-1395)	567 (87-1204)	0.775
Allantoic acid	1291 (349-20688)	2528 (1208-3925)	2556 (297-12194)	2294 (69-5513)	676 (203-12054)	3618 (203-18695)	0.612
Alanine-alanine	23001 (3539-51158)	35093 (8366-62578)	32801 (18769-42797)	26798 (1296-58300)	8220 (3554-39380)	15032 (6418-35642)	0.187
Alanine	831117 (330551-1023260)	621490 (238931-1333994)	767646 (433697-1574813)	787990 (108161-1343621)	497857 (248645-834572)	450923 (354139-2433628)	0.962
Adipic acid	1683 (624-3121)	1852 (906-3312)	2286 (1381-3483)	1592 (621-3252)	1543 (655-2615)	1371 (616-3171)	0.445
Adenosine	327 (242-1324)	241 (109-1923)	439 (133-987)	718 (33-1110)	308 (115-592)	347 (93-702)	0.370
Adenine	11511 (4811-31288)	13224 (4898-48286)	10140 (3842-26194)	13019 (836-35430)	14777 (4539-21084)	14449 (9953-19102)	0.928
Acetophenone NIST	12002 (1398-107094)	3716 (1684-4453)	7210 (2460-64181)	5865 (454-51902)	2757 (1583-136281)	4361 (1493-127060)	0.291
6-hydroxynicotinic acid	926 (344-1117)	1065 (412-16703)	3384 (355-20808)	1681 (440-8463)	1087 (114-4756)	451 (288-1392)	**0.012**
6-deoxyglucose	18381 (7866-34952)	13811 (6304-22976)	25267 (14248-52996)	19083 (3866-75563)	13808 (6551-17822)	10800 (2542-29994)	**0.038**
5-methoxytryptamine	1943 (670-4757)	759 (530-4757)	759 (530-2546)	920 (60-3085)	1079 (172-4125)	892 (135-4278)	0.473
5-aminovaleric acid	302773 (23299-373486)	313664 (129046-615614)	291535 (244586-801390)	252124 (107425-633659)	174921 (51974-741693)	317222 (46673-417913)	0.220
5,6-dihydrouracil	298 (91-1865)	413 (152-5865)	1181 (275-2157)	590 (62-1777)	487 (71-1848)	225 (55-2966)	0.563
4-pyridoxic acid	513 (319-1303)	397 (191-747)	327 (238-1139)	360 (140-698)	379 (152-904)	432 (201-1111)	0.486
4-methyl-5-thiazoleethanol	7363 (2218-13408)	4672 (1935-10524)	7386 (2602-14773)	5410 (358-18898)	2249 (1962-7795)	3721 (2770-8831)	0.881
4-hydroxyphenylacetic acid	30251 (12365-77357)	48378 (9868-185802)	80809 (2852-210032)	94999 (4712-349285)	2700 (2333-114656)	20099 (5735-244251)	0.335
4-hydroxyhippuric acid NIST	584 (148-816)	269 (100-724)	287 (77-940)	203 (49-440)	153 (50-578)	193 (45-1317)	0.565
4-hydroxybutyric acid	1528 (627-8541)	5021 (166-21943)	2716 (240-11446)	2265 (294-25408)	18241 (499-5024)	2384 (537-16733)	0.710
4-hydroxybenzoate	26015 (1126-46736)	15253 (8678-47897)	27781 (3851-44142)	29094 (439-52859)	8288 (1945-14124)	11132 (2850-23100)	0.371
4-aminobutyric acid	6266 (1136-19405)	4310 (2278-5541)	5142 (2518-9962)	5176 (606-16031)	4716 (1233-29774)	5119 (2542-18170)	0.578
3-phenyllactic acid	2410 (559-21974)	1548 (269-8496)	1696 (883-12797)	1213 (91-10116)	1121 (199-7057)	1195 (550-10915)	0.363
3-hydroxyphenylacetic acid	2488 (170-4390)	5443 (207-19918)	18788 (396-28085)	5798 (247-23697)	6646 (2447-22912)	4953 (1594-13876)	0.052
3-hydroxybutyric acid	4893 (1269-9691)	8579 (1464-44324)	17911 (2090-44034)	8151 (1459-18864)	6049 (3572-10258)	3272 (1765-15943)	0.136
3-hydroxybenzoic acid	254 (108-1018)	223 (49-663)	556 (343-971)	547 (61-731)	252 (95-550)	239 (149-1244)	0.071
3-hydroxy-3-methylglutaric acid	124 (55-530)	74 (55-792)	120 (84-340)	116 (55-360)	74 (43-303)	171 (75-649)	0.895
3-aminoisobutyric acid	5170 (1699-10093)	2412 (897-8087)	2817 (1629-41625)	3482 (1125-30785)	2978 (393-10547)	2473 (395-3169)	0.457

3,6-anhydro-D-galactose	1544 (760-1925)	1120 (891-2031)	1665 (692-2723)	1615 (157-2798)	886 (301-1281)	1564 (534-2353)	0.223
3,4-dihydroxyphenylacetic acid	1535 (325-3759)	639 (196-4054)	2498 (40-4435)	960 (24-5838)	1720 (40-2488)	1014 (156-3505)	0.340
3,4-dihydroxyhydrocinnamic acid NIST	20273 (6779-26330)	21373 (2095-272991)	12615 (12568-81738)	30435 (726-66369)	6920 (2489-13624)	9525 (625-46850)	0.931
3,4-dihydroxybenzoic acid	2360 (685-10262)	1576 (314-3119)	1812 (283-2131)	1336 (42-1620)	788 (144-805)	819 (346-1820)	0.083
3-(4-hydroxyphenyl) propionic acid	51287 (23153-223665)	112910 (25536-308951)	101777 (59055-139335)	71310 (37835-182873)	59372 (34663-177412)	77702 (4690-179661)	0.557
3-(3-hydroxyphenyl) propionic acid	190827 (46444-277436)	99761 (25694-347037)	242695 (161990-306432)	188674 (19918-259881)	101584 (2739-203863)	174725 (46444-277436)	0.485
3-(3-hydroxyphenyl)-3-hydroxypropionic acid NIST	297 (228-794)	256 (14-970)	1190 (208-1575)	902 (35-1292)	554 (31-1155)	296 (61-1232)	0.073
2-monopalmitin	9853 (4454-21311)	4815 (1432-19770)	5940 (1365-48320)	7370 (1966-16375)	4928 (2854-5586)	6661 (5683-20120)	0.022
2-monoolein	27048 (8061-60070)	11254 (3491-71801)	36954 (5871-158148)	11651 (77-34983)	9279 (178-17725)	21732 (950-47357)	0.917
2-methylglyceric acid NIST	987 (413-7486)	1320 (419-5471)	238 (138-2033)	203 (49-1252)	176 (65-767)	518 (186-1144)	**0.022**
2-hydroxyvaleric acid	1424 (664-3072)	1289 (615-2663)	1641 (1043-3524)	1545 (590-2327)	912 (522-2199)	1130 (202-1877)	0.103
2-hydroxyhexanoic acid	1305 (646-47408)	1259 (170-8249)	1106 (355-21461)	1461 (98-7060)	749 (161-5566)	1077 (545-16054)	0.671
2-hydroxyglutaric acid	735 (248-4252)	1313 (279-5468)	1070 (369-2611)	1557 (377-2478)	608 (377-2615)	1570 (325-15654)	0.338
2-hydroxybutanoic acid	2800 (1554-47108)	3895 (486-8277)	2200 (761-15634)	3853 (1062-8374)	859 (520-4603)	3139 (395-14965)	0.478
2-deoxytetronic acid	738 (280-1291)	822 (302-1524)	531 (393-1980)	636 (196-1239)	396 (202-441)	626 (388-1343)	0.172
2-deoxyerythritol	1862 (820-6902)	3888 (1056-9026)	3968 (1265-8124)	2837 (530-4602)	1230 (244-2138)	1703 (848-5221)	0.069
2,5-dihydroxypyrazine NIST	705 (501-1470)	1018 (277-1952)	823 (296-1750)	497 (332-1774)	922 (269-3856)	598 (309-1312)	0.811
2,4-diaminobutyric acid	3783 (1929-10413)	7591 (1000-15096)	11589 (4958-34975)	7517 (4770-11859)	2372 (977-14732)	2771 (1950-9542)	**0.019**
2,3-dihydroxybutanoic acid NIST	183 (30-560)	288 (46-351)	160 (28-489)	149 (40-224)	40 (28-330)	154 (48-338)	0.635
1-monostearin	1842 (334-2818)	1654 (610-3879)	1610 (367-4095)	2095 (173-5639)	1805 (484-2337)	835 (240-2927)	0.298
1-monopalmitin	6365 (1125-9506)	2814 (1528-5012)	5690 (858-10813)	3607 (198-7633)	971 (135-2070)	2142 (446-8116)	0.631
1-monoolein	13506 (12713-42907)	8874 (1313-47048)	14413 (4507-75044)	5496 (171-24063)	6572 (130-15604)	14163 (593-21650)	0.623
1-methylhydantoin	3986 (2310-22340)	4911 (3178-90204)	6578 (1953-129973)	2944 (1343-9973)	5030 (1190-112372)	4145 (2140-45714)	0.369
1-hexadecanol	2375 (1173-14652)	1231 (379-3840)	3061 (1112-9776)	1484 (128-11908)	1157 (486-2527)	3323 (181-4069)	0.724
1,5-anhydroglucitol	4837 (3378-6587)	3924 (2582-4814)	4695 (855-34620)	1902 (207-4897)	696 (91-3242)	3564 (1454-5655)	0.202
1,3-diaminopropane	5830 (542-10079)	4991 (1184-9070)	5099 (2327-8262)	5527 (349-8409)	1677 (761-8826)	3466 (2186-7978)	0.834
